# Signals Regulating Adhesion Dynamics

**DOI:** 10.1155/2012/785196

**Published:** 2012-12-31

**Authors:** Donna J. Webb, Claire M. Brown, Kris A. DeMali

**Affiliations:** ^1^Departments of Biological Sciences and Cancer Biology, Vanderbilt University, Nashville, TN 37235, USA; ^2^Department of Physiology, McGill University, Montreal, QC, Canada H3G 0B1; ^3^Department of Biochemistry, The University of Iowa, Iowa City, IA 52242, USA

Adhesions are sites of contact between cells (cell-cell) and between cells and the extracellular matrix (cell-ECM) that are essential for numerous biological processes such as embryogenesis, wound healing, and the immune response. They are composed of many different molecules including adhesion receptors, signaling proteins (e.g., kinases, phosphatases, and adaptor proteins), and structural proteins (e.g., actin-binding proteins) [1–3]. Adhesions are dynamic structures whose molecular composition and structure can undergo rapid changes to allow cells to respond to external signals [1, 4]. Indeed, this dynamic nature of adhesions within localized regions of cells is critical for many complex processes such as cell migration. 

For cell-ECM adhesions, their assembly is initiated by the binding of integrin adhesion receptors to the ECM. These new adhesions can either disassemble, allowing cells to migrate, or continue to mature by recruiting signaling and structural proteins to these sites. The dynamics and composition of adhesions are controlled by signaling networks that function to integrate molecular signals from outside and within cells. This special issue focuses on the molecular signals that regulate adhesion dynamics with an emphasis on cell-ECM adhesions. 

Integrins are transmembrane adhesion receptors that provide a functional link between the ECM and the actin cytoskeleton. Integrin engagement of the ECM serves to transduce signals to the interior of cells which regulate cell behavior. RGD-dependent integrins are a subgroup of adhesion receptors that specifically recognize the RGD motif, which is a three-amino-acid sequence (Arg-Gly-Asp) that is found in some ECM proteins. In this special issue, Y. D. Benoit et al. review recent findings regarding the importance of RGD-dependent integrins in epithelial cell homeostasis. Integrins also play important roles in blood clotting and wound healing by regulating platelet adhesion and aggregation. G. F. Guidetti and M. Torti discuss recent insights into the role of Rap1 in regulating platelet adhesion. Rap1 is a member of the Rap family of small GTPases and is a downstream effector of integrin signaling. A-Kinase-Anchoring Proteins (AKAP) are emerging as pivotal scaffolding proteins that bring together key signaling molecules to modulate cell migration. S. Akakura and I. H. Gelman provide insight into the regulation of AKAP12 and how it contributes to cell adhesion. Moreover, they discuss the scaffolding function of AKAP12 and its contribution to cell migration, maintenance of cytoskeletal architecture, cell proliferation, and cytokinesis. 

While integrin-containing matrix contacts were first identified in classic focal adhesions, they have subsequently been shown to exist in other structures including podosomes and invadopodia. Podosomes and invadopodia do not have the typical elongated shape of focal adhesions but form circular adhesive structures. Podosomes are found in phagocytic cells, while invadopodia are found in cancer cells, where as their name implies, they play a critical role in cancer cell invasion into the surrounding stroma. Focal adhesions, podosomes, and invadopodia share some of the same proteins, but they differ in their basic structure and function. P. P. Eleniste and A. Bruzzaniti compare and contrast the organization and function of focal adhesions, podosomes, and invadopodia. They highlight critical tyrosine kinases and signaling proteins that regulate the assembly and function of these adhesive structures. 

Cytotoxic necrotizing factors (CNF) are a class of autotransporter toxins that are made by uropathogenic *E. coli* (CNF1-3) and *Y. pseudotuberculosis* (CNF*γ*). CNF toxins deamidate and thus constitutively activate RhoA, Rac1, and Cdc42, which are critical regulators of adhesion dynamics. In this special issue, M. May et al. analyze the effects of CNF on cell-matrix adhesion. They show that CNF1 and CNF*γ* increase cell-matrix adhesion, resulting in reduced migration. They further indicate that the augmented cell-matrix adhesion is dependent on RhoA deamidation.

Adherens junctions are cell-cell adhesion sites that are important for the establishment and maintenance of apico-basal polarity of epithelial cells. C. Bertocchi et al. give an overview of the kinases and phosphatases that regulate the phosphorylation of adherens junction proteins. In addition, they discuss the phosphorylation events that control the assembly and disassembly of adherens junctions. 

Overall, this special issue is a reflection of the diverse roles (e.g., epithelial cell homeostasis to the immune response to cancer) that adhesions play in biological processes and diseases. This issue provides an overview of the different types of adhesive structures, including focal adhesions, adherens junctions, and podosome as well as the molecular composition, regulation, and dynamics of these structures. The goal of this special issue is to provide the reader with an appreciation of the importance and function of adhesions and a synopsis of key signaling molecules that comprise adhesions.

*Donna J. Webb* *Donna J. Webb* *Claire M. Brown* *Claire M. Brown* *Kris A. DeMali* *Kris A. DeMali*
